# Post-acute pathways among hip fracture patients: a system-level analysis

**DOI:** 10.1186/s12913-016-1524-1

**Published:** 2016-07-18

**Authors:** Kristen B. Pitzul, Walter P. Wodchis, Michael W. Carter, Hans J. Kreder, Jennifer Voth, Susan B. Jaglal

**Affiliations:** Institute of Health Policy, Management, and Evaluation, University of Toronto, 160-500 University Avenue, Toronto, Ontario M5G1V7 Canada; Institute for Clinical Evaluative Sciences, 155 College Street, Suite 425, Toronto, Ontario M5T3M6 Canada; Toronto Rehabilitation Institute, University Health Network, 160-500 University Avenue, Toronto, Ontario M561V7 Canada; Department of Mechanical and Industrial Engineering, University of Toronto, 5 King’s College Road, Toronto, Ontario M5S3G8 Canada; Department of Surgery, University of Toronto, 2075 Bayview Avenue., MG-365, Toronto, Ontario M4N3M5 Canada; Department of Physical Therapy, University of Toronto, 160-500 University Avenue, Toronto, Ontario M5G1V7 Canada

**Keywords:** Hip fractures, Rehabilitation, Care pathways, Health system planning, Resource allocation, Regional variation

## Abstract

**Background:**

Hip fractures among older adults are one of the leading causes of hospitalization and result in significant morbidity, mortality, and health care use. Guidelines suggest that rehabilitation after surgery is imperative to return patients to pre-morbid function. However, post-acute care (which encompasses rehabilitation) is currently delivered in a multitude of settings, and there is a lack of evidence with regards to which hip fracture patients should use which post-acute settings. The *purpose* of this study is to describe hip fracture patient characteristics and the most common post-acute pathways within a 1-year episode of care, and to examine how these vary regionally within a health system.

**Methods:**

This study took place in the province of Ontario, Canada, which has 14 health regions and universal health coverage for all residents. Administrative health databases were used for analyses. Community-dwelling patients aged 66 and over admitted to an acute care hospital for hip fracture between April 2008 and March 2013 were identified. Patients’ post-acute destinations within each region were retrieved by linking patients’ records within various institutional databases using a unique encoded identifier. Post-acute pathways were then characterized by determining when each patient went to each post-acute destination within one year post-discharge from acute care. Differences in patient characteristics between regions were detected using standardized differences and *p*-values.

**Results:**

Thirty-six thousand twenty nine hip fracture patients were included. The study cohort was 71.9 % female with a mean age of 82.9 (±7.5SD). There was significant variation between regions with respect to the immediate post-acute discharge destination: four regions discharged a substantially higher proportion of their patients to inpatient rehabilitation compared to all others. However, the majority of patient characteristics between those four regions and all other regions did not significantly differ. There were 49 unique post-acute pathways taken by patients, with the largest proportion of patients admitted to either community-based or short-term institutionalized rehabilitation, regardless of region.

**Conclusions:**

The observation that similar hip fracture patients are discharged to different post-acute settings calls into question both the appropriateness of care delivered in the post-acute period and health system expenditures. As policy makers continue to develop performance-based funding models to increase accountability of institutions in the provision of quality care to hip fracture patients, ensuring patients receive appropriate rehabilitative care is a priority for health system planning.

**Electronic supplementary material:**

The online version of this article (doi:10.1186/s12913-016-1524-1) contains supplementary material, which is available to authorized users.

## Background

In 2000, there were approximately 1.6 million hip fractures worldwide and by 2050 this number is projected to increase to 6.26 million [1–5]. Hip fractures are most prevalent in elderly women with fragile bones with an underlying pathology of osteoporosis [1, 6–13]. Hip fractures often occur spontaneously or from minimal trauma (e.g., fall from standing height or less) [14].

Hip fractures result in extensive morbidity, mortality, and health care use. Between 30 and 50 % of patients do not return to their pre-morbid function even two years post-fracture and estimates of attributable mortality rates 1 year post-fracture range between 20 and 30 % [10, 15–17]. Hip fracture patients have three times the in-patient cost compared to age-matched non-hip fracture patients, and post-acute utilization is also substantial due to the requirement of rehabilitation after surgery [18–22]. From a Canadian health system perspective, 1-year attributable health-care cost of hip fractures is $1.1 billion [15].

Research from many countries has highlighted the need for the provision of evidence-based quality care for hip fracture patients [23–25]. To encourage delivery of quality care, some health systems (e.g., National Health System in England and Wales) have increased institutional accountability by implementing financial models tied to metrics for care delivered to hip fracture patients (i.e., performance-based funding) [23, 24, 26]. The focus of these funding models has been the acute care period, with quality measured by performance indicators (i.e., time to surgery) and evidence-based generalizable outcomes (e.g., 30-day re-admission rates) [23–25]. However, there is little research focused on the measurement of quality of care delivered in the post-acute period.

Previous evidence surrounding quality of care delivery in the post-acute period has focused on patient-level predictors of three outcome measures: morbidity (e.g., functional ability), mortality (e.g., in-hospital mortality), and post-acute health care use (e.g., 30-day re-admission rates). Age and cognitive impairment are well established predictors of both morbidity and mortality. Other well established predictors include gender and pre-morbid functional ability (which predict post-fracture morbidity); co-morbid conditions (which predict mortality and post-fracture and health care use); frailty (which predicts mortality); and previous health care use (which predicts post-fracture health care use) [25, 27–31]. [32–36]. [28, 29, 37–39]. History of falls and previous fractures are suggested as important considerations for care delivery, although their exact role is unknown [25, 28, 40, 41]. Measuring these patient-level predictors is important to anticipate patients’ outcomes after fracture, however, without knowing where these patients should receive care, for example inpatient rehabilitation or a community setting, stakeholders lack a comprehensive understanding of which settings would optimize patient outcomes. There is currently little evidence surrounding which hip fracture patients should use which health care resources in the post-acute period.

Characterizing variations of historical and/or current practice patterns in health care use is an important step in determining which patients should use which health care resources [42–44]. By characterizing these variations, insight is gained into what future research is needed for improving effectiveness and/or efficiency or how best to implement policy [42–46]. Describing care pathways throughout the health care system is one approach for determining geographic variations in the use of health system resources [47]. Patient pathways are dependent on both patient population characteristics and health system structure (e.g., care settings) [47, 48].

In the literature, the characterization of pathways for hip fracture patients is extremely limited and is focused solely on the immediate discharge destination after an acute care stay [49–51]. Increased age, presence of co-morbidities, and a history of dementia all increase the probability of being immediately discharged to a skilled nursing facility or long-term care institution [49, 52]. Pre-morbid living condition (i.e., institutionalization versus non-institutionalization) is also a predictor of post-acute discharge setting after hip fracture repair [6, 29, 53]. Furthermore, evidence from the United States, England, and Canada suggests there is variation, even within a single health region, in the proportion of patients that are immediately discharged to each post-acute setting [50, 51, 54]. There are currently no studies that describe hip fracture care pathways or patient characteristics after this immediate discharge destination and throughout the entire first year after a hip fracture (which is considered to be the length of time that health care use can be attributable to the hip fracture) [15, 55].

In order to support the development of evidence-based recommendations for quality care, determining which hip fracture patients use which post-acute resources is imperative. The *purpose* of this study is to describe hip fracture patient characteristics and post-acute care pathways in the first year following a hip fracture, and to examine how these pathways vary regionally within a health system.

## Methods

### Overall approach

**Fig. 1:**
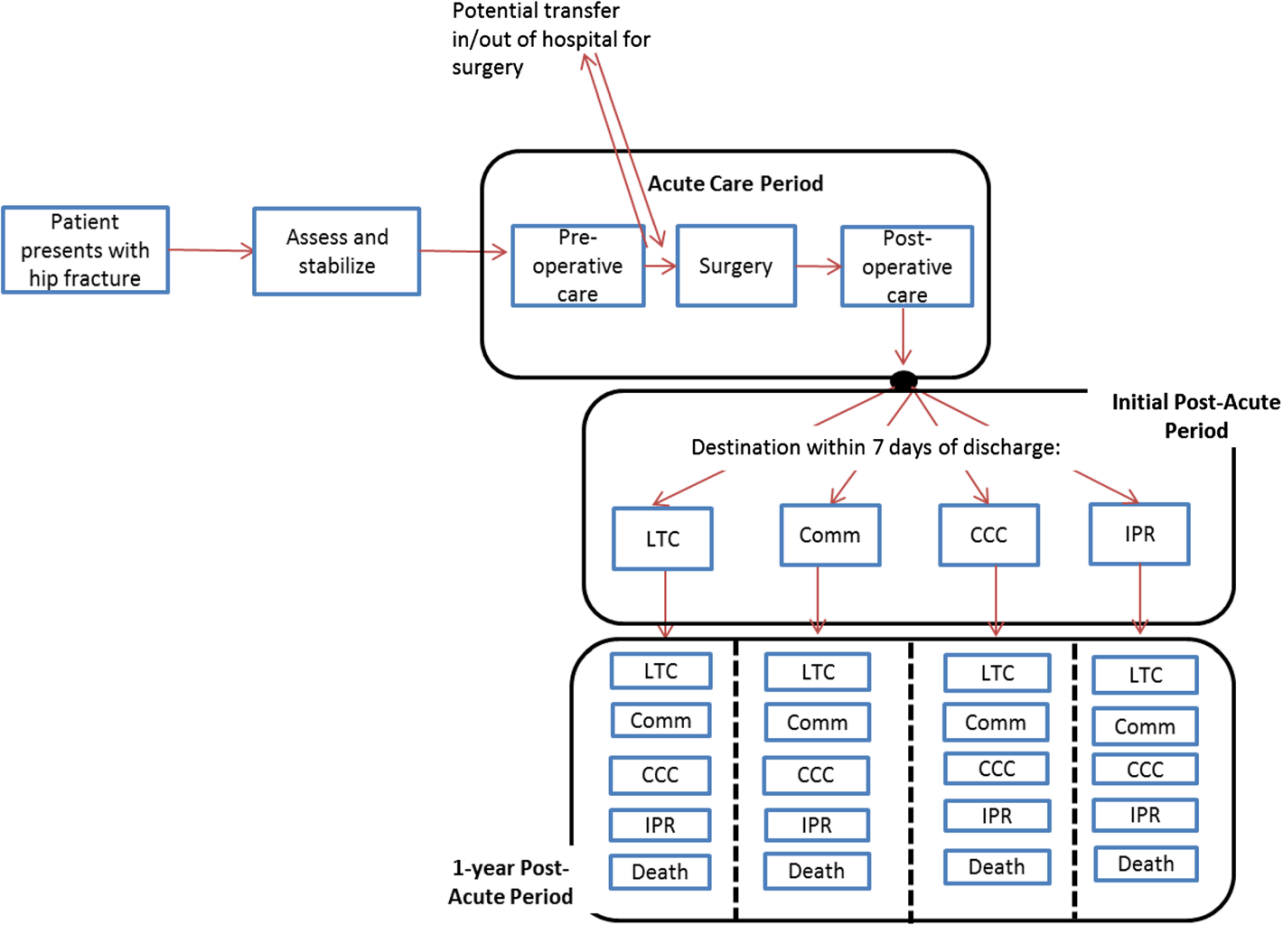
The 1-year episode of care for hip fracture patients in Ontario, Canada. LTC = Long-Term Care; Comm = Community; CCC = Complex Continuing Care; IPR = Inpatient Rehabilitation; HC = Home Care

This was a retrospective cohort study completed from a health system perspective in the province of Ontario, Canada. For the purposes of administering health care delivery, Ontario is divided into 14 health regions [56]. Provincial-level databases were used to obtain patients’ demographic and clinical information to characterize which hip fracture patients experience which post-acute care pathways within 1-year post-fracture in each health region (Fig. 1). The date of study index was defined as the date of admission to acute care for hip fracture.

### Study setting

Ontario is Canada’s most populous province, with both urban and rural communities and provincially-funded health care for all 13 million residents [57–59]. The 14 health regions vary in the number of people served, funding distribution, and the number of health care institutions, but all health regions possess the following post-acute care destinations: Long-term care facilities (LTC); complex continuing care facilities (CCC) for longer-term rehabilitation (i.e., over 30 days) or patients with complex medical conditions; inpatient rehabilitation facilities (IPR) for short-term rehabilitation (i.e., 30 days or less); and home care (HC), which may include home-based rehabilitation (HBR) (Additional file 1: Table S1) [24, 58].

### Data sources

All patient-level data were analyzed at the Institute for Clinical Evaluative Sciences (ICES), a prescribed entity funded by the Ontario Ministry of Health and Long-Term Care (MOHLTC) [60]. ICES holds provincial-level databases that contain population (e.g., age) and geographic information (e.g., county), as well as health records for patients within each publically-funded sector in Ontario’s health system (e.g., physician billings, inpatient care, and pharmaceutical costs for persons aged 65 and older) [60, 61]. Each patient has a unique coded identifier that can be linked between databases to capture patient information throughout the entire health system.

Patient demographic information (i.e., patients’ health region, birth year, sex, and rurality, and date of death) was retrieved from the Registered Persons Database (RPDB) and the 2006 Ontario Census (i.e., patients’ income) [62]. Acute care inpatient records (i.e., admission rates, re-admission rates, and co-morbidities) were captured using the Discharge Abstracts Database (DAD) and reasons for emergency department visits (i.e., previous fractures, previous malignant neoplasm, falls, co-morbidities) were captured using the National Ambulatory Care Reporting System (NACRS) [61]. All relevant demographic and clinical variables are defined below. Admissions to LTC and CCC were identified using the Complex Continuing Reporting System (CCRS), IPR records were captured by the National Rehabilitation Reporting System (NRS), and HC records were identified using the Ontario Association of Community Care Access Centre’s home care database (HCD) [61]. The Ontario Health Insurance Claims database (OHIP) was used to capture physician billing information to help capture co-morbidity information [61, 63].

Information on health regions’ resource availability (e.g., number of inpatient rehabilitation beds) was obtained from the MOHLTC’s Health Data Web Branch Portal, which collects information on financial and other activity measures for organizations within each health region [64].

### Study cohort

Patients admitted to acute care between April 1^st^ 2008 and March 31^st^ 2013 with a most responsible diagnosis of hip fracture were identified using International Classification of Disease Version 10 diagnosis codes (ICD10CA) S72.08, S72.09, S72.10, S72.19, or S72.20. Using diagnosis codes in administrative databases is a valid and reliable method for capturing hip fracture patients [65–67]. Patients’ who were not Ontario residents, were missing sex or age or were older than 105 years of age were excluded. Patients who were younger than 66 years of age, had a pathological fracture (ICD10CA M8445) or Paget’s disease (ICD10CA M880, M881, M888), or who were institutionalized pre-fracture were excluded because they were deemed to represent atypical hip fracture patients [15].

### Definitions of demographic and clinical characteristics

A number of demographic and clinical variables that are either theoretically important or have previously been shown to be important when describing health care utilization are defined below. These characteristics were used to compare hip fracture patients both between health regions and between immediate post-acute discharge destinations.

#### Demographic variables

Demographic characteristics included patient’s age, sex, health region, rurality [28, 30, 37, 68, 69]. Patient’s age at index was calculated by subtracting the patient’s birth year from the patient’s acute care admission date. Patient health region and sex were captured verbatim by the RPDB. Rurality was calculated using the 2008 Rurality Index of Ontario (RIO2008) [70]. The RIO2008 is a score designed to measure a person’s community (i.e., region based on partial postal code) population density and accessibility to basic and advanced health care [70]. It is reported as an ordinal scale from 0 to 100, with higher numbers indicating greater rurality [70]. The Ontario mean RIO2008 score was 45.2 [70]. The percentage of communities more urban than the provincial average was therefore calculated by determining the number of communities with a RIO2008 less than 45.2 [70]. Income quintiles were derived by linking mean household income within a given geographic area (from Ontario Census) with the patients’ postal codes, which is a known and valid method of estimating socioeconomic status [28, 71, 72].

#### Clinical variables prior to index admission

Clinical characteristics prior to index admission were collected to provide insight into patients’ morbidity upon admission to acute care and included; previous falls, previous diagnosis of malignant neoplasm, previous fragility fractures, Charlson comorbidity score, John Hopkin’s Aggregated Diagnosis Groups© (ADGs), and pre-fracture home care use. All characteristics were collected within 1 year prior to index unless otherwise stated and are described below.

Falls resulting in an emergency department visit or acute care admission were captured using ICD10CA codes for any diagnosis type (ICD10CA W00, W01, W04-W08) [27]. Similarly, a diagnosis of malignant neoplasm was defined using ICD10CA codes for any diagnosis type upon emergency department visit or acute care admission (ICD10CA C0-C9) [15]. Previous fragility fractures (humerus, wrist, spine, and hip) were captured using ICD10 codes for any diagnosis upon admission to acute care or emergency department visit (ICD10CA_humerus_ S42.2; ICD10CA_wrist_ S62.0-S62.2; ICD10CA_spinal_ S32.0; ICD10CA_hip_ S72.08, S72.09, S72.10, S72.19, or S72.20) [27].

Two different indices of co-morbidities were calculated: Charlson Score, and John Hopkin’s Aggregated Diagnosis Groups (ADGs) [73–77]. Charlson Score is a well-established predictor of mortality in the hip fracture population and the ADG system is a more recent co-morbidity index that has been shown to be a strong predictor of mortality in older adults [35, 78–82]. Each patient is assigned to one or more ADGs to a maximum of 32 ADGs per patient [83]. An increasing number of ADGs indicates increased co-morbidity [83]. For the purposes of this study, patients within the lower 25 % (0 to 3 ADGs) is reported as “lower co-morbidity burden”; and the proportion of patients in the top 75 % (i.e., more than 8 ADGs) is reported as “higher co-morbidity burden” with the middle population included as a reference category. Both measures of co-morbidity were captured using relevant ICD10CAs from acute care admissions.

The proportion of patients who had home care pre-fracture was determined by linking the study cohort to home care records and determining which patients had a home care service date within 1 year prior to index admission [73].

#### Clinical variables during index and after admission

Prevalent complications and co-morbid conditions that arose during index acute care admission were captured using ICD10CA codes. These conditions included: Delirium (ICD10CA F050, F051, F058, F059) or dementia (ICD10CA F013, F018, F019, F030); and malignant neoplasm ((ICD10CA C0-C9) [29, 30, 37–39, 50, 84, 85]. Risk factors for frailty were also collected (possession of 1 or more is a known indicator of frailty): Chronic kidney disease (ICD10CA N181-N185, N189); acidosis (ICD10CA E872); liver cirrhosis (ICD10CA K743-K746); diabetes (ICD10CA E10-E13); chronic heart failure (ICD10CA I50); peripheral arterial disease (ICD10CA I739); COPD (ICD10CA J40-J44) [73]. The proportion of patients’ who died in-hospital was calculated by determining which patients had death dates between their index acute care admission date and acute care discharge date.

The functional independence measure (FIM) contains 18 items that measures a patient’s disability level and ability to perform activities of daily living [86]. FIM scores range from 18 (lowest level of functioning) to 126 (highest level of functioning) [86]. In Ontario, hospitals with IPR beds are required to complete a FIM score within 72 h of patient admission, therefore FIM admission score can be used as a proxy indicator of patient’s functional ability upon discharge to acute care [87, 88].

### Post-acute care pathways

Post-acute care pathways were examined for hip fracture patient’s 1 year episode of care (Fig. 1). The characterization of post-acute care pathways was a complete case analysis, meaning that patients who died in-hospital or within 7 days of discharge from acute care were excluded.

Immediate discharge destination to LTC, CCC, or IPR was defined as an admission date to the institution of interest within 7 days of index acute care discharge date [24]. Immediate discharge destination to community was defined as an absence of any admission dates to LTC, CCC, or IPR within 7 days of discharge from index acute care. The modal number of days between acute care discharge and admission to the immediate discharge destination was determined to ensure that 7 days was an appropriate (i.e., inclusive) definition for immediate discharge destination. After determining this immediate discharge destination, the remaining pathways were characterized by linking the study cohort to LTC, CCC, and IPR records 1-year post-acute care discharge and determining the number of days between admission date(s) and index acute care discharge date. Patients’ health care use could therefore be ordered from closest to index acute care discharge to furthest, enabling characterization of care pathways. Patients who died prior to any given admission were removed from the cohort so that calculated proportions are based only on alive patients (i.e., patients with a potential for further health care use).

Patients were considered to be living in the community when no admission date was available for any institution. The proportion of community-dwelling patients who had home care or home-based rehabilitation was determined by linking these patients to HCD. Home-based rehabilitation was defined as at least one visit with a physiotherapist or occupational therapist [89].

### Statistical analyses

Statistical differences in hip fracture patient characteristics between health regions and immediate discharge destinations were determined using Chi-Square tests (categorical variables), Fisher’s exact test (categorical variables with value of less than 6), or Analysis of variance (ANOVA; continuous variables) where appropriate. Significance was defined as comparisons with an associated *p*-value of less than 0.001. For comparisons involving only two groups (i.e., by grouped health region), standardized differences were used instead of *p*-values to detect differences in effect size A standardized difference of 0.200 or greater was considered significant [90].

## Results

**Fig. 2:**
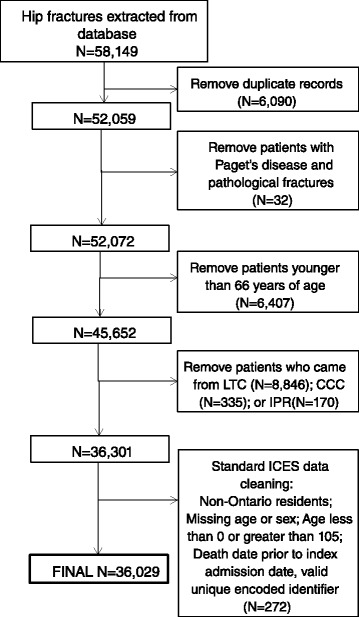
Application of exclusion criteria and data cleaning to cohort of patients admitted for hip fracture in Ontario, April 2008-March 2013. LTC = Long-term care; CCC = complex continuing care; IPR = inpatient rehabilitation

A total of 52,059 hip fractures were identified as having an index acute care admission within the study timeframe. After data cleaning and application of exclusion criteria, 36,029 unique hip fracture patients were included (Fig. 2).

The study cohort of hip fracture patients were representative of a typical hip fracture patient population, with a mean age of 82.9 (±7.5) and 64.6 % over the age of 81 years (Table 1) [15]. Most patients were females (71.9 %) and living in communities considered more urban than the provincial average (Table 1). Within one year pre-fracture, a small proportion of patients had fragility fractures (3.1 %) but almost half of patients had a fall(s) (42.3 %) (Table 1). Most patients had none or few co-morbid conditions, with over half (52.8 %) of patients with a Charlson Score of 0, and 32.2 % of patients were considered to have a lower co-morbidity burden according to ADGs (Table 1). Only slight clinical differences in patient characteristics existed between the health regions (Additional file 1: Table S2).

**Table 1:** Characteristics of persons admitted to acute carefor hip fracture and of those discharged alive by immediate discharge destination (e.g., LTC)

		Acute care	LTC	CCC	IPR	Community	*P*-value
Total N		36,029	1,943	5,492	13,994	11,920	.
*Demographic Characteristics*							
Mean (±SD) Age at index		82.88 ± 7.5	85.20 ± 6.99	84.40 ± 6.92	82.73 ± 7.13	81.28 ± 7.85	<.001
81 years or older at index, n (%N)		23,294 (64.6 %)	1,475 (75.9 %)	4,003 (72.9 %)	9,049 (64.7 %)	6,642 (55.7 %)	<.001
Female sex, n (%N)		25,930 (71.9 %)	1,521 (78.3 %)	3,953 (72.0 %)	10,339 (73.9 %)	8,573 (71.9 %)	<.001
More urban than provincial average n (%N)		33,415 (92.7 %)	1,955 (97.8 %)	5,487 (96.1 %)	13,667 (97.9 %)	11,910 (85.6 %)	<.001
Income Quintile, n (%N)	1 (lowest income)	7,710 (21.4 %)	361 (18.6 %)	1,191 (21.7 %)	2,383 (17.0 %)	3,213 (27.0 %)	<.001
	2	7,329 (20.3 %)	398 (20.5 %)	1,048 (19.1 %)	2,963 (21.2 %)	2,381 (20.0 %)	
	3	7,641 (21.2 %)	481 (24.8 %)	1,270 (23.1 %)	3,244 (23.2 %)	2,023 (17.0 %)	
	4	6,525 (18.1 %)	372 (19.1 %)	970 (17.7 %)	2,722 (19.5 %)	1,926 (16.2 %)	
	5 (highest income)	6,682 (18.5 %)	323 (16.6 %)	995 (18.1 %)	2,640 (18.9 %)	2,288 (19.2 %)	
*Clinical characteristics prior to index*							
Previous hip fracture (1991-), n (%N)		2,405 (6.7 %)	176 (9.1 %)	354 (6.4 %)	818 (5.8 %)	837 (7.0 %)	<.001
Any fragility fracture 1 year prior, n (%N)		1,123 (3.1 %)	79 (4.1 %)	198 (3.6 %)	433 (3.1 %)	433 (3.6 %)	0.03
Falls 1 year prior, n(%N)		15,244 (42.3 %)	886 (45.6 %)	2,607 (47.5 %)	6,137 (43.9 %)	5,614 (47.1 %)	<.001
Malignant neoplasm 1 year prior, n (%N)		1,593 (4.4 %)	61 (3.1 %)	271 (4.9 %)	493 (3.5 %)	538 (4.5 %)	<.001
Charlson score (grouped), n (%N)	0	18,078 (52.8 %)	916 (49.5 %)	2,755 (52.0 %)	7,364 (56.7 %)	5,446 (56.9 %)	<.001
	1	8,285 (24.2 %)	491 (26.6 %)	1,300 (24.5 %)	3,082 (23.7 %)	2,216 (23.2 %)	
	2	4,295 (12.6 %)	253 (13.7 %)	695 (13.1 %)	1,515 (11.7 %)	1,090 (11.4 %)	
	3+	3,554 (10.4 %)	189 (10.2 %)	552 (10.4 %)	1,038 (8.0 %)	815 (8.5 %)	
Number of ADG Groups 1 year prior, n (%N)	lower co-morbidity burden	11,615 (32.2 %)	608 (31.3 %)	1,680 (30.6 %)	4,442 (31.7 %)	4,072 (34.2 %)	<.001
	median co-morbidity burden	14,326 (39.7 %)	795 (40.9 %)	2,139 (38.9 %)	5,739 (41.0 %)	4,688 (39.3 %)	
	higher co-morbidity burden	10,107 (28.0 %)	540 (27.8 %)	1,673 (30.5 %)	3,813 (27.2 %)	3,160 (26.5 %)	
Had home care 1 year prior, n(%N)		10,434 (28.9 %)	713 (36.7 %)	1631 (29.7 %)	2,491 (17.8 %)	2,244 (18.7 %)	<.001
*Clinical characteristics during index*							
Diagnosis of delirium, n (%N)		1,519 (4.4 %)	114 (6.2 %)	277 (5.2 %)	462 (3.6 %)	362 (3.8 %)	<.001
Diagnosis of dementia, n (%N)		2,549 (7.5 %)	195 (10.5 %)	435 (8.2 %)	750 (5.8 %)	699 (7.3 %)	<.001
Diagnosis of pressure ulcer, n (%N)		911 (2.7 %)	74 (4.0 %)	162 (3.1 %)	263 (2.0 %)	221 (2.3 %)	<.001
Diagnosis of malignant neoplasm, n (%N)		1,426 (4.0 %)	68 (3.5 %)	247 (4.5 %)	405 (2.9 %)	463 (3.9 %)	<.001
Frailty risk factors >1, n (%N)		2,680 (7.4 %)	96 (5.2 %)	290 (5.5 %)	521 (4.0 %)	385 (4.0 %)	<.001

### Immediate post-acute discharge destinations

For description of post-acute care pathways, 2,680 patients (7.4 %) who died in-hospital were excluded, resulting in 33,349 hip fracture patients included in the following analyses. Hip fracture patients discharged to IPR or community were younger, had a lower proportion of home care usage prior to index, and had slightly lower co-morbidity burden compared to hip fracture patients discharged to LTC or CCC (Table 1).

**Fig. 3:**
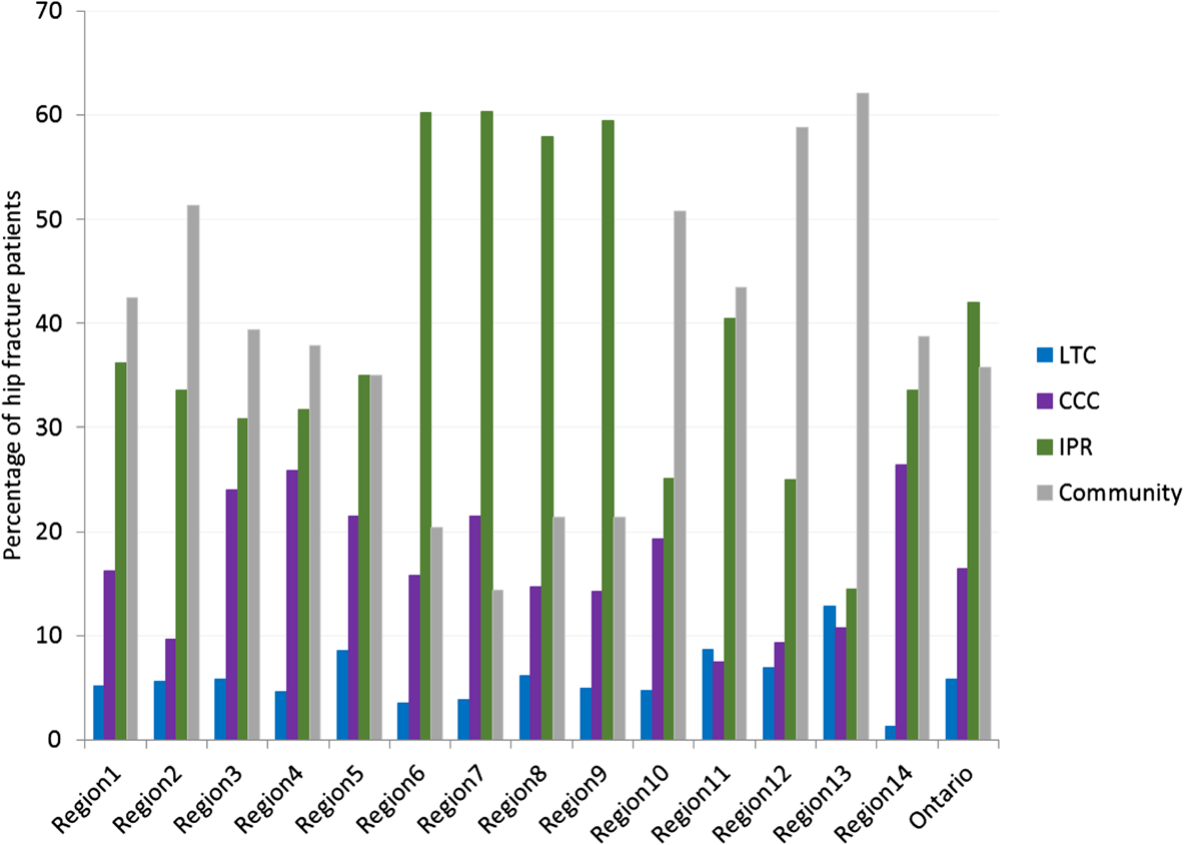
Percentage of hip fracture patients admitted to long-term care (LTC), complex continuing care (CCC), inpatient rehabilitation (IPR), or to the community within 7 days of discharge from index acute care visit, by health region, fiscal 2008–2013

Despite consistency in baseline hip fracture patient characteristics, there was variation between health regions in the immediate post-acute discharge destinations (Fig. 3). Health region 6-health region 9 discharged a substantially higher proportion of their patients to IPR (between 57.9 and 60.4 %) compared to all other health regions (between 14.4 and 40.5 %) (Fig. 3). This trend was consistent regardless of year of index acute care admission (Additional file 2: Figure S1).

A few hip fracture patient characteristics in health regions6-9 (i.e., High-IPR health regions significantly differed from patient characteristics in other health regions (Table 2). Other regions have a lower proportion of patients living in urban areas and within the highest income quintile compared to High-IPR health regions, however this difference can be attributed to the health regions characteristics (i.e., High-IPR health regions encompass large urban centers, with a large proportion of wealth) as opposed to differences between hip fracture patients. However, all other regions also havea slightly lower proportion of patients with higher co-morbidity, and with acute care delirium diagnosis compared to health regions6-9 (Table 2). Furthermore, when patients were grouped by immediate discharge destination and characteristics (including mean or median admission functional independence measure (FIM)) were compared between health regions6-9 and all other health regions, there were a few significant differences found (Table 3). Clinical characteristics varied the most between health regions6-9 and other regions for patients discharged to LTC: there were a lower proportion of patients that have a diagnosis of delirium and diagnosis of malignant neoplasm 1 year prior to index in all other regions compared to HighIPR regions (Table 3). For patients discharged to IPR, those in other regions had significantly higher FIM admission scores compared to health regions6-9. It should be noted, however, that the minimal clinical important difference in FIM score has not yet been established in hip fracture patients (Table 3). There were no differences between health regions6-9 and other regions for patients discharged to the community (Table 3). There is also variation in resource use between High-IPR health regions and all other health regions: High-IPR health regions spend slightly less per patient on home care services, have more IPR and CCC beds, but less LTC beds per 10,000 persons aged 66 and over compared to all other health regions (Additional file 1: Table S3).

**Table 2:** Characteristics of persons discharged from acute care for hip fracture by high-IPR regions and other regions

		High-IPR	Others	Ontario	SD
Number of patients with hip fracture discharged from acute care		12,470	20,879	33,349	.
*Demographic Characteristics*					
Mean (±SD) age at index		82.90 ± 7.39	82.47 ± 7.50	82.63 ± 7.46	0.06
81 years or older at index, n(%N)		8,112 (65.1 %)	13,057 (62.5 %)	21,169 (63.5 %)	0.05
Female, n(%N)		9,074 (72.8 %)	15,312 (73.3 %)	24,386 (73.1 %)	0.02
More urban than provincial average n(%N)		12,320 (98.8 %)	18,770 (89.9 %)	31,081 (93.2 %)	0.36
Income Quintiles, n(%N)	1 (lowest income)	2,016 (16.2 %)	5,132 (24.6 %)	7,148 (21.4 %)	0.26
	2	2,647 (21.2 %)	4,143 (19.8 %)	6,790 (20.4 %)	
	3	2,232 (17.9 %)	4,786 (22.9 %)	7,018 (21.0 %)	
	4	2,520 (20.2 %)	3,470 (16.6 %)	5,990 (18.0 %)	
	5 (highest income)	3,014 (24.2 %)	3,232 (15.5 %)	6,246 (18.7 %)	
*Clinical Characteristics prior to index*					
Previous hip fracture (1991-), n(%N)		808 (6.5 %)	1,377 (6.6 %)	2,185 (6.6 %)	0.01
Any fragility fracture 1 year prior, n(%N)		457 (3.7 %)	686 (3.3 %)	1,143 (3.4 %)	0.02
Falls 1 year prior, n(%N)		5,536 (44.4 %)	9,708 (46.5 %)	15,244 (45.7 %)	0.04
Malignant neoplasm 1 year prior, n(%N)		490 (3.9 %)	873 (4.2 %)	1,363 (4.1 %)	0.08
Grouped Charlson Score, n(%N)	0	7,583 (65.8 %)	12,581 (68.1 %)	20,164 (67.2 %)	0.03
	1	2,168 (18.8 %)	3,244 (17.6 %)	5,412 (18.0 %)	
	2	1,027 (8.9 %)	1,541 (8.3 %)	2,568 (8.6 %)	
	3+	750 (6.5 %)	1,109 (6.0 %)	1,859 (6.2 %)	
Number of ADG groups 1 year prior, n(%N)	lower co-morbidity burden	3,738 (30.0 %)	7,064 (33.8 %)	10,802 (32.4 %)	0.11
	median co-morbidity burden	4,987 (40.0 %)	8,374 (40.1 %)	13,361 (40.1 %)	
	higher co-morbidity burden	3,745 (30.0 %)	5,441 (26.1 %)	9,186 (27.5 %)	
Had home care 1 year prior, n(%N)		9,801 (78.6 %)	15,844 (79 %)	26,312 (78.9 %)	0.01
*Clinical Characteristics at index*					
Diagnosis of delirium, n(%N)		851 (7.4 %)	1,075 (5.8 %)	1,926 (6.4 %)	0.11
Diagnosis of dementia, n(%N)		468 (4.1 %)	759 (4.1 %)	1,227 (4.1 %)	0.04
Diagnosis of malignant neoplasm, n(%N)		402 (3.2 %)	781 (3.7 %)	1,183 (3.5 %)	0.03
Frailty risk factors >1, n(%N)		1,105 (9.6 %)	1,690 (9.1 %)	2,795 (9.3 %)	0.01

**Table 3:** Characteristics of hip fracture patients by immediate discharge destination, high-IPR regions and other regions

		High-IPR	Other	SD	High-IPR	Other	SD	High-IPR	Other	SD	High-IPR	Other	SD
Immediate Discharge Destination^a^		LTC			CCC			IPR			Community		
Number of patients		1,919	935	.	3,299	5,252	.	7,398	6,596	.	3,435	12,718	.
*Demographic Characteristics*													
Mean (+SD) age at index		83.90 ± 7.15	83.79 ± 7.61	0.02	83.48 ± 7.42	83.23 ± 7.30	0.03	82.55 ± 7.18	82.94 ± 7.05	0.02	82.78 ± 7.75	82.18 ± 7.63	0.08
81 years or older at index, n(%N)		1,338 (69.7 %)	652 (69.7 %)	0.00	2,239 (67.9 %)	3,474 (66.1 %)	0.04	4,699 (63.5 %)	4,350 (65.9 %)	0.01	2,208 (64.3 %)	7,728 (60.8 %)	0.07
Female, n(%N)		1,447 (75.4 %)	682 (72.9 %)	0.06	2,314 (70.1 %)	3,777 (71.9 %)	0.04	5,445 (73.6 %)	4,894 (74.2 %)	0.01	2,411 (70.2 %)	9,168 (72.1 %)	0.04
More urban than provincial average n(%N)		1,835 (95.6 %)	878 (93.9 %)	0.12	3,124 (94.7 %)	4,907 (93.4 %)	0.05	7,265 (98.2 %)	6,372 (96.6 %)	0.01	171 (5.0 %)	1,321 (10.4 %)	0.19
Income Quintiles, n(%N)	1 (lowest income)	389 (20.3 %)	165 (17.6 %)	0.18	581 (17.6 %)	1,210 (23.0 %)	0.14	1,160 (15.7 %)	1,223 (18.5 %)	0.08	742 (21.6 %)	3,110 (24.5 %)	0.06
	2	425 (22.1 %)	167 (17.9 %)		681 (20.6 %)	1,002 (19.1 %)		1,660 (22.4 %)	1,303 (19.8 %)		763 (22.2 %)	2,557 (20.1 %)	
	3	463 (24.1 %)	208 (22.2 %)		692 (21.0 %)	1,202 (22.9 %)		1,369 (18.5 %)	1,875 (28.4 %)		631 (18.4 %)	2,489 (19.6 %)	
	4	340 (17.7 %)	190 (20.3 %)		622 (18.9 %)	948 (18.1 %)		1,454 (19.7 %)	1,268 (19.2 %)		590 (17.2 %)	2,186 (17.2 %)	
	5 (highest income)	290 (15.1 %)	201 (21.5 %)		706 (21.4 %)	870 (16.6 %)		1,728 (23.4 %)	912 (13.8 %)		699 (20.3 %)	2,305 (18.1 %)	
*Clinical Characteristics prior to index*													
Previous hip fracture (1991-), n(%N)		127 (6.6 %)	80 (8.6 %)	0.01	218 (6.6 %)	353 (6.7 %)	0.00	431 (5.8 %)	387 (5.9 %)	0.01	248 (7.2 %)	823 (6.5 %)	0.03
Any fragility fracture 1 year prior, n(%N)		57 (3.0 %)	37 (4.0 %)	0.06	124 (3.8 %)	180 (3.4 %)	0.02	259 (3.5 %)	174 (2.6 %)	0.00	107 (3.1 %)	466 (3.7 %)	0.03
Falls 1 year prior, n(%N)		850 (44.3 %)	443 (47.4 %)	0.06	1,616 (49.0 %)	2,419 (46.1 %)	0.02	3,203 (43.3 %)	2,934 (44.5 %)	0.01	1,580 (46.0 %)	5,950 (46.8 %)	0.02
Malignant neoplasm 1 year prior, n(%N)		69 (3.6 %)	26 (2.8 %)	0.13	111 (3.4 %)	216 (4.1 %)	0.01	259 (3.5 %)	234 (3.5 %)	0.01	154 (4.5 %)	598 (4.7 %)	0.01
Grouped Charlson Score, n(%N)	0	919 (47.9 %)	420 (44.9 %)	0.00	1,595 (48.3 %)	2,703 (51.5 %)	0.06	4,650 (66.9 %)	4,173 (68.1 %)	0.00	1,800 (52.4 %)	7,026 (55.2 %)	0.06
	1	504 (26.3 %)	284 (30.4 %)		839 (25.4 %)	1,287 (24.5 %)		1,287 (18.5 %)	1,085 (17.7 %)		822 (23.9 %)	3,006 (23.6 %)	
	2	266 (13.9 %)	133 (14.2 %)		462 (14.0 %)	688 (13.1 %)		583 (8.4 %)	518 (8.5 %)		465 (13.5 %)	1,481 (11.6 %)	
	3+	230 (12.0 %)	98 (10.5 %)		403 (12.2 %)	574 (10.9 %)		435 (6.3 %)	349 (5.7 %)		348 (10.1 %)	1,205 (9.5 %)	
Number of ADG groups 1 year prior, n(%N)	lower co-morbidity burden	571 (29.8 %)	228 (24.4 %)	0.09	797 (24.2 %)	1,415 (26.9 %)	0.07	2,268 (30.7 %)	2,174 (33.0 %)	0.04	844 (24.6 %)	3,313 (26.0 %)	0.04
	median co-morbidity burden	779 (40.6 %)	413 (44.2 %)		1,376 (41.7 %)	2,187 (41.6 %)		2,983 (40.3 %)	2,756 (41.8 %)		1,462 (42.6 %)	5,439 (42.8 %)	
	higher co-morbidity burden	569 (29.7 %)	294 (31.4 %)		1,126 (34.1 %)	1,650 (31.4 %)		2,147 (29.0 %)	1,666 (25.3 %)		1,129 (32.9 %)	3,966 (31.2 %)	
Had home care 1 year prior, n(%N)		751 (39.1 %)	355 (38.0 %)	0.02	1,047 (31.7 %)	1,691 (32.2 %)	0.01	1092 (14.8 %)	906 (13.7 %)	0.02	1,030 (30.0 %)	3,419 (26.9 %)	0.07
*Clinical Characteristics during and after index*													
Diagnosis of delirium, n(%N)		110 (5.7 %)	50 (5.3 %)	0.14	221 (6.7 %)	243 (4.6 %)	0.09	391 (5.6 %)	297 (4.8 %)	0.07	187 (5.4 %)	451 (3.5 %)	0.08
Diagnosis of dementia, n(%N)		194 (10.1 %)	119 (12.7 %)	0.08	319 (9.7 %)	406 (7.7 %)	0.07	270 (3.9 %)	235 (3.8 %)	0.04	310 (9.0 %)	875 (6.9 %)	0.08
Diagnosis of malignant neoplasm, n(%N)		70 (3.6 %)	28 (3.0 %)	0.03	111 (3.4 %)	216 (4.1 %)	0.04	200 (2.7 %)	205 (3.1 %)	0.01	141 (4.1 %)	538 (4.2 %)	0.01
Frailty risk factors >1, n(%N)		113 (5.9 %)	54 (5.8 %)	0.00	223 (6.8 %)	310 (5.9 %)	0.04	643 (9.2 %)	526 (8.6 %)	0.04	173 (5.0 %)	617 (4.9 %)	0.01
Mean (±SD) FIM admission		.	.	.	.	.	.	73.36 ± 17.1	75.65 ± 15.8	0.16			
Median (IQR) FIM admission		.	.	.	.	.	.	76 (63–86)	78 (66–87)				

^a^ *LTC* long term care, *CCC* complex continuing care, *IPR* inpatient rehabilitation

### Post-acute care pathways

**Fig. 4:**
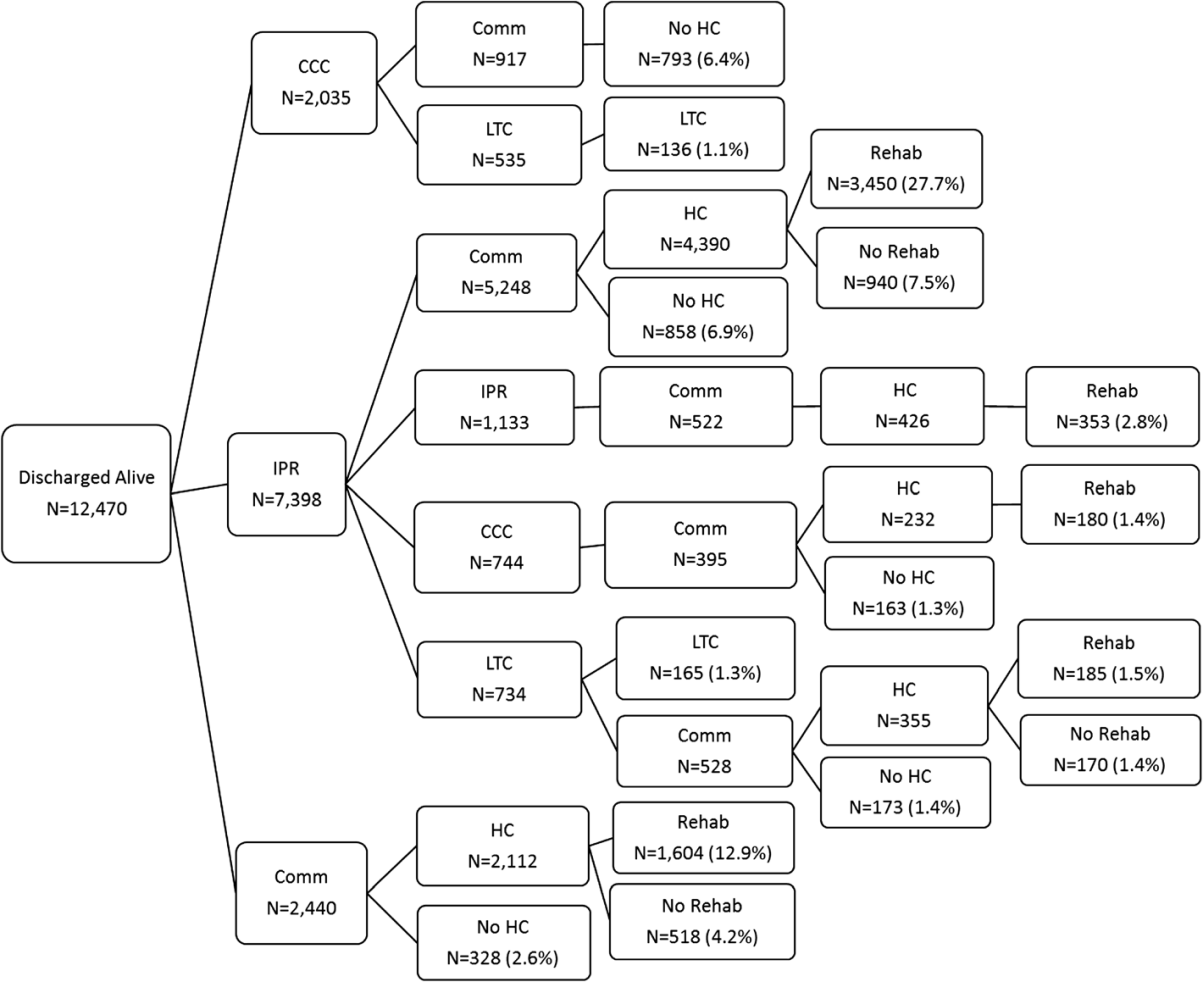
Post-acute pathways for 80 % of hip fracture patients discharged from acute care between April 1st 2008 to March 31st 2013 in HighIPR regions. The number of patients at each destination within a pathway (including patients who died) is recorded along with the percentage of total patients that underwent the entire pathway. For example, 917 patients are discharged to CCC and then discharged to the community. Of these 917 patients, 793 do not receive home care, therefore 6.4 % of total patients undergo the pathway: CCC-COMM-NO HC. By far the most common pathway (27.7 % of total patients) is IPR-COMM-HC-REHAB. CCC = complex continuing care; IPR = inpatient rehabilitation; Comm = community; HC = home care; Rehab = home-based rehabilitation

**Fig. 5:**
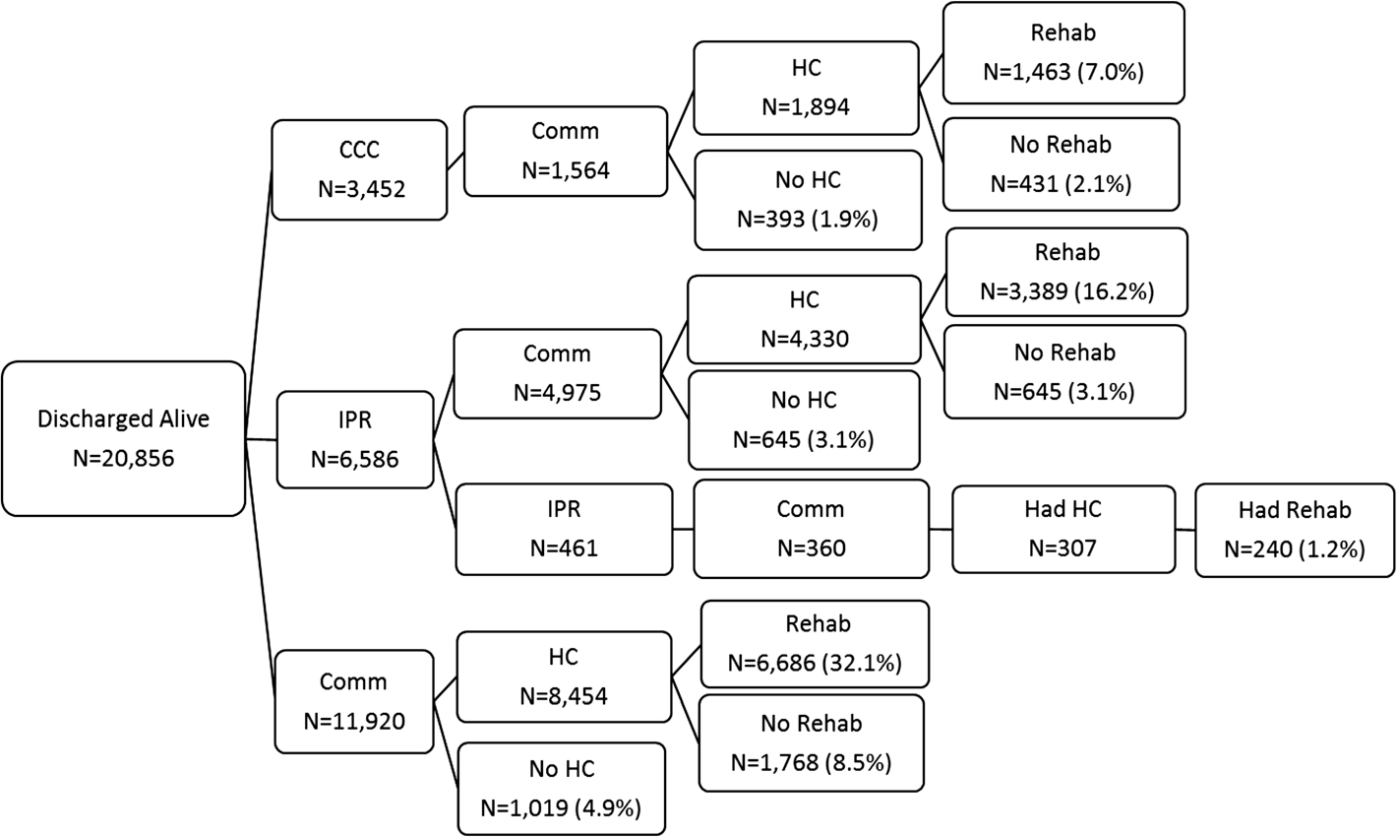
Post-acute pathways for 80 % of hip fracture patients discharged from acute care between April 1st 2008 to March 31st 2013 in Other regions. The number of patients at each destination within a pathway (including patients who died) is recorded along with the percentage of total patients that underwent the entire pathway. For example, 1,564 patients are discharged to CCC and then discharged to the community. Of these 1,564 patients, 393 do not receive home care, therefore 1.9 % of total patients undergo the pathway: CCC-COMM-NO HC. By far the most common pathway (32.1 % of total patients) is COMM-HC-REHAB. CCC = complex continuing care; IPR = inpatient rehabilitation; Comm = community; HC = home care; Rehab = home-based rehabilitation

**Fig. 6:**
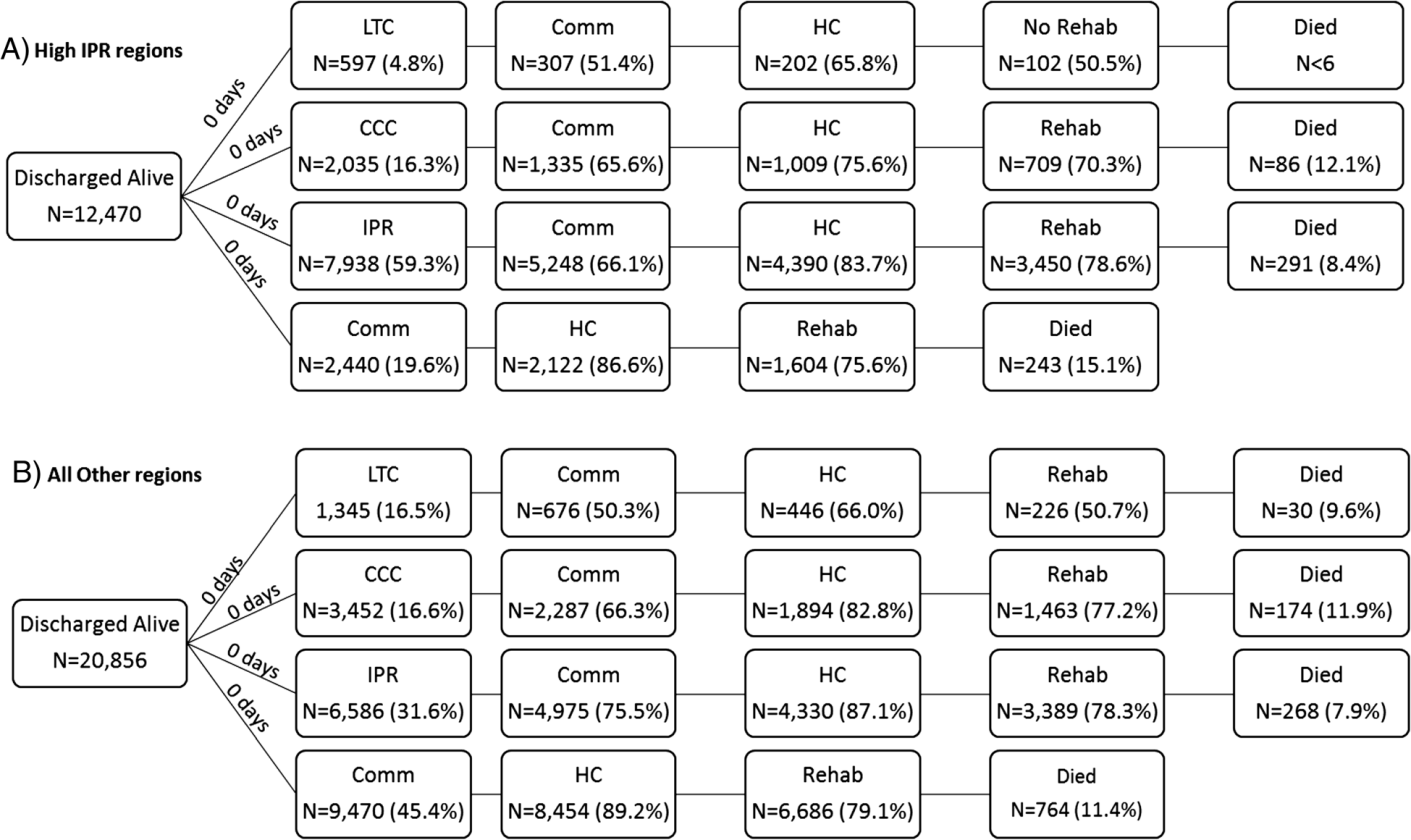
Post-acute pathways for hip fracture patients in **a** High-IPR regions, and **b** all other regions, April 1st 2008-March 31st 2013. LTC = long-term care; CCC = complex continuing care; IPR = inpatient rehabilitation; Comm = community; HC Service = home care service; Rehab = home-based rehabilitation. Reading from left to right is the proportion of patients immediately discharged to each post-acute destination, followed by the most common destinations (i.e., the destinations taken by the largest proportion of patients). However, other pathways can be deduced by subtracting what is shown (i.e., the numerator) from the denominator (e.g., in high-IPR LHINs, 307 patients went from LTC to community, of which 202 had home care service. Therefore, 105 patients who went from LTC to community did not have home care service). The modal number of days that each patient was discharged to CCC, IPR, or LTC, respectively, was 0 days. The proportions of patients who died in each pathway are also shown. Note that the patients who died in the less common trajectories are not depicted

Forty-nine unique post-acute care pathways were found for hip fracture patients in Ontario (Additional file 3: Figure S2). However, 80 % of hip fracture patients undergo 1 of 15 pathways in High-IPR or 1 of 10 pathways in all other regions (Figs. 4 and 5). The most common pathway by far (27.7 % of total) for patients in High-IPR regions is an immediate discharge destination of inpatient rehabilitation followed by community–based rehabilitation (Fig. 4). Contrarily, the most common pathway (32.1 % of total) for patients in all other regions is an immediate discharge destination to the community followed by community-based rehabilitation (Fig. 5). When the most common pathways for each immediate discharge destination within High-IPR regions and all other regions are examined, 7 out of 8 of the pathways included community-based rehabilitation, regardless of immediate discharge destination (Fig. 6). Therefore the most common pathways differed between High-IPR health regions and all other health regions primarily with regards to immediate discharge destination: in addition to differences in the proportion of patients immediately discharged to IPR described above, High-IPR health regions also have a lower proportion of patients immediately discharged to LTC (4.8 %) compared to all other health regions (16.5 %; Fig. 6). Regardless of health region, the modal number of days between acute care discharge and admission to immediate post-discharge destination was 0, therefore the use of 7 days as a cut-off is considered inclusive.

When the most common pathways by immediate discharge destination are compared within the two grouped health regions, it is clear that a larger proportion of patients died when discharged directly to the community followed by home-based rehabilitation (15.1 and 11.4 % respectively) compared to patients discharged directly to IPR followed by community-based rehabilitation (8.4 and 7.9 % respectively)) (Fig. 6).

## Discussion

This study describes hip fracture patients and their post-acute care pathways for 1-year post-fracture and examined variations in care pathways across health regions within the province of Ontario, Canada. In four of the health regions (i.e., high-IPR health regions), there were larger proportions of patients discharged directly to IPR and smaller proportions discharged directly to the community and to long-term care relative to all other health regions (i.e., other regions). This variation exists despite similarity in most clinical characteristics between high-IPR and other regions. In both High-IPR regions and other regions, approximately 50 % of patients undergo one of two pathways: Discharged to IPR followed by community-based rehabilitation (i.e., IPR pathway), or discharged directly to community followed by community-based rehabilitation (i.e., community pathway). Interestingly, within both high-IPR regions and other regions, a higher proportion of patients who underwent the community pathway die compared to patients discharged directly to the IPR pathway. Lastly, regardless of health region or immediate discharge destination, the largest proportion of hip fracture patients end up living in the community with home-based rehabilitation within 1 year-post fracture.

There are few published studies that describe geographic variation in the proportion of hip fracture patients immediately discharged to IPR or the community [50, 51, 54]. Neuburger et al. [54] found that within one geographical area in the United Kingdom, there was wide variation (between 2.1 and 54.7 %) in hospital discharge practices with regards to the proportion of hip fracture patients discharged to community rehabilitation. Results from our study had much smaller variation between health regions, with a range of approximately 17 to 40 % of patients discharged to the community with home care services. Similarly, Maciejewski et al. [50] study of United States veterans with a hip fracture had a much smaller proportion of patients discharged to inpatient rehabilitation (16.9 %) compared to the proportion found in the current study for both high-IPR health regions and other health regions. Another study from the United Kingdom by Drew et al. [51] found that there was significant variation even within one service organization (i.e., eight acute care sites), with rehabilitation being provided in both inpatient settings and outpatient settings. Coupled with the difference within high-IPR regions and other regions in the proportion of patients who underwent the IPR pathway compared to the community pathway is the difference in proportion of patients who died between these two pathways. Although mortality is an important patient outcome, and these differences should be explored further, it should be noted that the relationship presented in this study between a patient’s care pathway and mortality is not necessarily causal.

The geographic variation in post-acute resource use by hip fracture patients in this study is not surprising given the minimal and conflicting evidence about which rehabilitation setting is appropriate for which hip fracture patients. Furthermore, because patient care pathways are impacted by not only population characteristics (e.g.,co-morbid conditions), but health system structure (e.g., availability of beds), it is difficult to determine why the variation in immediate discharge destination exists. The higher proportion of patients discharged immediately to LTC in other health regions compared to High-IPR health regions may be an example of the impact of health system structure (i.e., availability of beds) on health care use (i.e., use of LTC): all other health regions had more LTC beds than high-IPR health regions. Similarly, high-IPR health regions have more IPR beds compared to all other health regions. Further research is required to determine the impact of this variability in post-discharge destinations on patient outcomes. It is clear that without evidence, there is not only a lack of guidance for the allocation of post-acute discharge settings by acute care clinicians, but stakeholders have no guide for prioritizing the provision of certain resources.

Regardless of health region or immediate discharge destination, the final destination for the largest proportion of hip fracture patients in this study is rehabilitation provided in the community. A 2013 review of the effectiveness of inpatient versus community rehabilitation settings in hip fracture patients yielded mixed results, with most evidence graded as low or moderate quality, and the effectiveness of either setting being dependent on how effectiveness was measured [91]. Edgren et al. [92] randomized controlled trial on the impact of a home-based rehabilitation program on physical disability in hip fracture patients (compared to standard care) concluded that a larger sample size was needed to confirm results. Similarly, Latham et al. [93] randomized controlled trial on the effectiveness of a home exercise program on hip fracture patient functional ability concluded that more research is needed to determine if there is clinical relevance to their findings. Considering this evidence and the results of this study suggesting the large proportion of hip fracture patients using community-based rehabilitation, future work should focus on the effectiveness of community-based rehabilitation for hip fracture patients.

This study has limitations. First, despite the power of large administrative databases, variables are collected for purposes other than research and may therefore be incomplete or of weak validity [94]. Second, although as many relevant patient demographic and clinical characteristics were collected as possible, there were some (e.g., patient discharge preferences) that were unavailable. There may therefore have been some differences or reasons for differences between high-IPR health region and other health region patient characteristics that are unknown. Finally, although the hip fracture patient cohort, post-acute discharge settings, and health policies in Ontario are similar to other public-payer systems, because patient care pathways are dependent on health system structure, the generalizability of results to certain health systems (i.e., multi-payer private systems) may be limited.

The notion that similar hip fracture patients are discharged to different post-acute settings calls into question both the appropriateness of care delivered in the post-acute period and health system expenditures. If different settings yield similar quality of care and outcomes (i.e., effectiveness) for similar patients, then resources should be allocated such that the less expensive setting is prioritized, particularly within a public payer system. If different settings result in differences in effectiveness, then there is likely an inequity in the provision of post-acute resources. Results from this study, in a similar fashion to recent studies regarding the provision of rehabilitation to hip fracture patients, support the need for an evidence-based approach to the appropriate allocation of rehabilitation settings for hip fracture patients [51, 89, 95].

## Conclusion

As policy makers continue to develop performance-based funding models to increase accountability of institutions in the provision of quality care to hip fracture patients, ensuring patients receive appropriate rehabilitative care is a priority for health system planning. Future research should focus on determining the costs and effectiveness of post-acute care pathways in hip fracture patients using comparative effectiveness analyses in order to develop recommendations for the delivery of evidence-based quality care across the entire continuum.

## Abbreviations

ADG, John Hopkins’ Aggregated Diagnosis Groups; CCC, complex continuing care; CCRS, continuing care reporting system; DAD, discharge abstracts database; DINs, drug identification numbers; FIM, functional independence measure; HBR, home-based rehabilitation; HC, home care; HCD, home care database; health region, local health integration network; ICD-10, International classification of disease code version 10; ICES, Institute for clinical evaluative sciences; IPR, inpatient rehabilitation; LTC, long-term care; MOHLTC, Ministry of Health and long-term care; NACRS, National Ambulatory Care Reporting System; NRS, National rehabilitation reporting system; OHIP, Ontario Health Insurance claims database; RIO2008, rurality index of Ontario; RPDB, registered persons database

## Additional files

Additional file 1: Table S1. Epidemiology and resource indicators for all health regions in Ontario, fiscal 2008–2013. **Table S2**. Demographic and clinical characteristics of persons admitted to acute care for surgical repair of hip fracture in Ontario, by health region, fiscal 2008–2013. **Table S3**. Epidemiology and resource indicators for High IPR LHINs and all other LHINs, Ontario, fiscal 2008-2013. (XLSX 29 kb) Additional file 2: Figure S1. Percentage of hip fracture patients admitted to long-term care (LTC), complex continuing care (CCC), inpatient rehabilitation (IPR), or to the community within 7 days of discharge from index acute care visit, by health region, fiscal 2008–2013. (PDF 132 kb) Additional file 3: Figure S2. All possible post-acute care pathways for hip fracture patients in Ontario, fiscal 2008–2013. (PDF 263 kb)
