# Supplementary material for: Post-acute pathways among hip fracture patients: a system-level analysis
Source: BMC Health Serv Res. 2016 Jul 18;16:275. doi: 10.1186/s12913-016-1524-1 (PMC4950780; doi:10.1186/s12913-016-1524-1)
Supplement: Additional file 2: Figure S1. — Percentage of hip fracture patients admitted to long-term care (LTC), complex continuing care (CCC), inpatient rehabilitation (IPR), or to the community within 7 days of discharge from index acute care visit, by health region, fiscal 2008–2013. (PDF 132 kb) [file 12913_2016_1524_MOESM2_ESM.pdf]

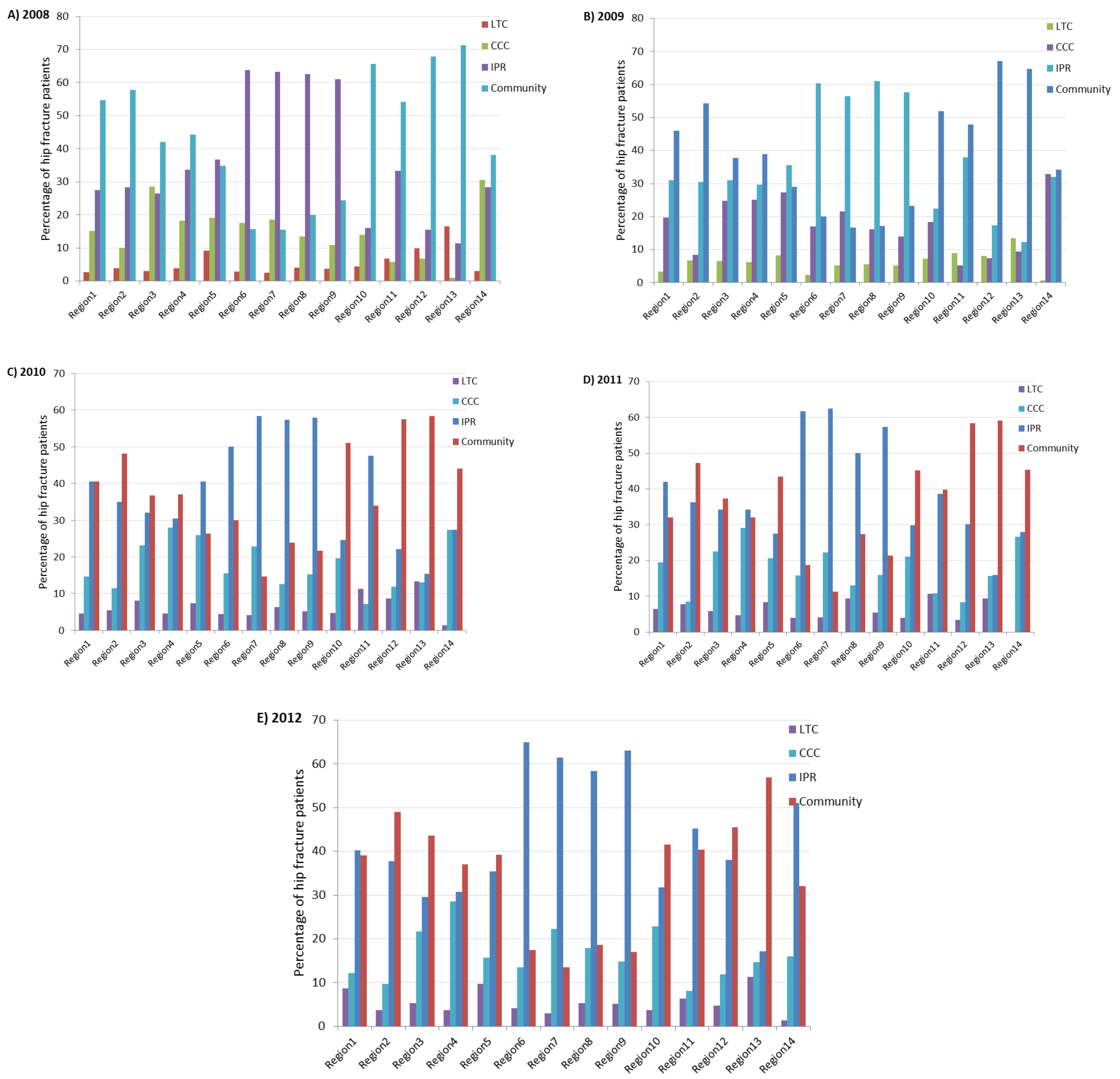

Figure 1SA-E. Percentage of hip fracture patients admitted to long-term care (LTC), complex continuing care (CCC), inpatient rehabilitation (IPR), or to the community within 7 days of discharge from index acute care visit, by health region (LHIN), fiscal 2008-2013.
