# Supplementary figures and images for: Post-acute pathways among hip fracture patients: a system-level analysis
Source: BMC Health Serv Res. 2016 Jul 18;16:275. doi: 10.1186/s12913-016-1524-1 (PMC4950780; doi:10.1186/s12913-016-1524-1)

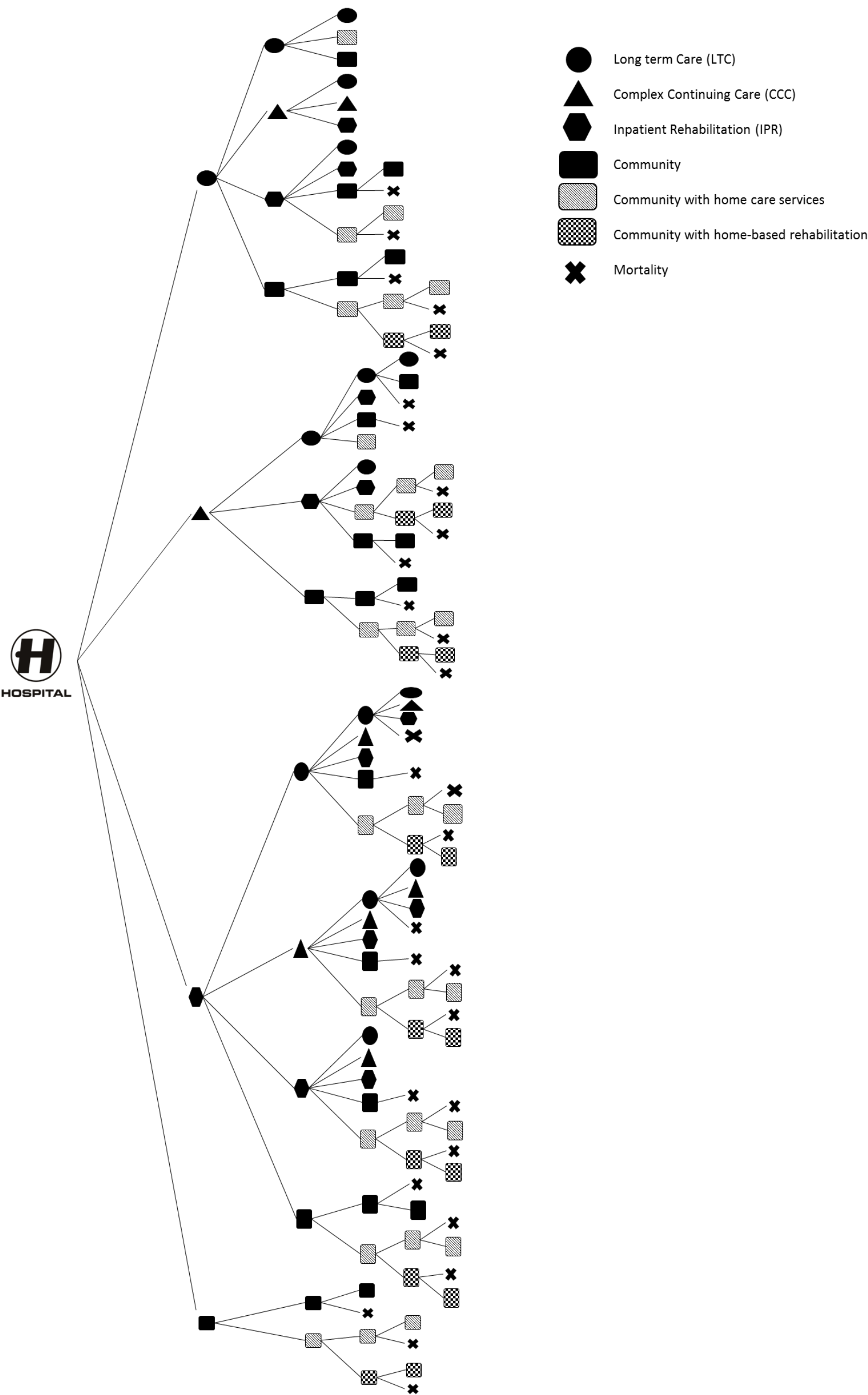

Figure 2S. All possible post-acute care pathways for hip fracture patients in Ontario, fiscal 2008-2013.

Supplement: Additional file 3: Figure S2. — All possible post-acute care pathways for hip fracture patients in Ontario, fiscal 2008–2013. (PDF 263 kb) [file 12913_2016_1524_MOESM3_ESM.pdf]
